# The clinical significance of some serum tumor markers among chronic patients with *Helicobacter pylori* infections in Ibb Governorate, Yemen

**DOI:** 10.1186/s13027-023-00542-7

**Published:** 2023-10-12

**Authors:** Marwan K. Saeed, B. A. Al-Ofairi, Mohammed A. Hassan, M. A. Al-Jahrani, Ahmed M. Abdulkareem

**Affiliations:** 1 Department of Biological Sciences, Microbiology Section, Faculty of Science, Sana’a University, Sana’a, Yemen; 2 Pathology Department, Faculty of Medicine, Taiz University, Taiz, Yemen; 3 Departement of Medical Laboratory, Faculty of Medical Sciences, Queen Arwa University, Sana’a, Yemen; 4 Department of Medical Laboratories, University of Science and Technology, Ibb, Yemen

**Keywords:** *Helicobacter pylori*, Tumor markers, Chronic *H. pylori* infections, Carcinoembryonic antigen, Cancer antigen 19-9, Caner antigen 72-4

## Abstract

**Background:**

*Helicobacter pylori* (*H. pylori*) is a carcinogenic bacterium, it is the greatest risk factor for gastric cancer (GC), according to these evidences, there may be a certain association between chronic *H. pylori* infections and serum levels of tumor markers. This study was conducted to determine serum levels of some tumor markers, namely carcinoembryonic antigen (CEA), cancer antigen 19-9 (CA19-9) and cancer antigen 72-4 (CA72-4) in patients with chronic *H. pylori* infections and evaluate the association between serum tumor marker levels and chronic patients with *H. pylori* infections in Ibb Governorate, Yemen.

**Subjects and methods:**

This study involved 200 patients who had been diagnosed with *H. pylori* infections using a serum immunochromatography antibody test. Stool and blood samples were collected from all patients to confirm the presence of *H. pylori* through detection of serum *H. pylori* IgG antibody and stool antigen test (SAT). Additionally, serum samples were analyzed to measurement the level of certain tumor markers CEA, CA19-9 and CA72-4. These tests were conducted at various Hospitals, Gastroenterology and Hepatology clinics in Ibb governorate, Yemen from October 2019 to November 2020.

**Results:**

The findings of current study showed that the prevalence of *H. pylori* infections by rapid anti *H. pylori* test were 200 (100%), 157 (78.5%) by serum *H. pylori* IgG antibody and 108 (54%) by SAT. In addition, the results showed that 42 (21%) of the patients had abnormal level of CEA, 30 (15%) had abnormal level of CA19-9 and 31 (15.5%) had abnormal level of CA72-4. Most importantly, the results indicated that the serum tumor marker levels CEA, CA19-9 and CA72-4 were correlated with the levels of serum *H. pylori* IgG antibody as well as positive results from the SAT (*P *< 0.05). Furthermore, the results indicated that serum tumor marker levels were associated with different infection status. Finally, the results indicated that the serum levels of tumor markers were associated with older ages, symptomatic patients and long duration of *H. pylori* infections (*P *< 0.05).

**Conclusion:**

The findings of this study indicated that there is a significant association between chronic *H. pylori* infections and the serum levels of tumor markers (CEA, CA19-9 and CA72-4). This suggests that the patients with active chronic *H. pylori* infection may have an increased risk of developing GC. Therefore, monitoring and early detection of *H. pylori* infection and tumor markers levels in these patients may be crucial for identifying individuals at higher risk and implementing appropriate interventions.

## Introduction

*H. pylori* is a Gram-negative, microaerophilic, rod-shaped bacterium that infects the gastric mucosa in more than half of the human population worldwide. Infections with this bacterium can lead to the development of several gastropathies such as chronic gastritis (CG), peptic ulcer (PU), atrophy and metaplasia. Additionally, *H. pylori* infection is considered as one of the main risk factors for the development of gastric adenocarcinoma. As a result, the International Agency for Research on Cancer (IARC) subsidized by the World Health Organization (WHO) has classified *H. pylori* bacterium as a type I carcinogen [1–3].

Virulence factors of *H. pylori* play a crucial role in the progression and outcomes of infection. Urease as an important virulence factor for *H. pylori* plays a role in pathogenicity establishment and ammonia production that disrupt the tight cell junctions, breaks cellular integrity, and damage the gastric epithelium. In addition to urease was recently reported to possibly contribute to tumor growth and metastatic dissemination by inducing angiogenesis and playing a key role in GC progression. Furthermore, the most studied molecule is the cytotoxin associated gene (CagA), which is translocated by the type IV secretion system of *H. pylori* into gastric cells, generating intracellular signals that facilitate malignancy. Individuals have an increased risk of developing GC if they express cagA+ instead of cagA−, and the strains of *H. pylori* that carry CagA are associated with an increased risk of developing CG or PU. Vacuolating cytotoxin A (VacA) is another virulence mechanism of *H. pylori* correlated with GC. One of the mechanisms attributed to VacA is its interference with Interleukin-2 production and Interleukin-2 receptor expression, which in turn reduces the proliferation of T lymphocytes [4–7].

*Helicobacter pylori* causes progressive damage to the gastric mucosa and plays a causative role in a number of important diseases, including duodenal ulcer (DU) disease; gastric ulcer (GU) disease, gastric adenocarcinoma and gastric mucosa associated lymphoid tissue (MALT) lymphoma. It is estimated that *H. pylori* positive patients have a 10–20% lifetime risk of developing ulcer disease and 1–2% risk of developing GC. Most patients with early GC have no obvious symptom. Some patients have symptoms of nausea, vomiting, or similar upper gastrointestinal tract that cannot be paid attention. Consequently, the diagnosis rate of early GC is relatively low. With disease development, patient has obvious symptoms of upper gastrointestinal discomfort such as abdominal pain, loss of appetite, weight loss, fatigue, nausea, vomiting, and suffocation. However, most patients had been in an advanced stage when the above symptoms occurred. At this stage, the prognosis is poor because of the difficulty of treatment [8–11].

GC is a common malignancy in the gastric mucosa and gastric glands in the digestive tract. It is the second highest cause of cancer deaths worldwide due to the high prevalence of *H. pylori* infections. The most recent reports from the IARC estimates that 78% of all GC are attributable to *H. pylori* infection. In addition seroprevalence studies estimate that 90% of GC patients have encountered *H. pylori* infections. Furthermore, *H. pylori* induces DNA damage and aberrant DNA methylation genome instability and mutation are emerging hallmarks of cancer development. *H. pylori* infection increases the risk of GC development by about two times. Most human GC develop after long-term *H. pylori* infections. The progression is extending to lymphoproliferative gastric lymphoma, and it is the most dangerous factor leading to GC [12–18]**.**

Tumor markers used in GC patients as prognostic factors and a close relationship with specific cancers such as CEA, CA19-9 and CA72-4 and cancer antigen 125, to play an important roles in predicting recurrence, metastasis and evaluating prognosis of GC [19–23]**.**

According to the evidences stated above, there seems to be a certain association between *H. pylori* infections and tumor markers, the higher levels of CEA in patients with *H. pylori* infections. These results indicated that *H. pylori* infections may play a role in the progression of specific cancer and has the potential to regard as a useful marker for cancer. The CEA, CA72-4 and CA19-9 were used for the diagnosis of patients with chronic infections with *H. pylori* infections, because several studies reported a strong association between chronic *H. pylori* infections with GC and colorectal cancer. As a result, *H. pylori* is now recognized as a Group 1 carcinogen for humans [24–26]. The current study was designed to: determine the serum levels of some tumor markers (CEA, CA19-9, CA72-4) in chronic patients with *H. pylori* infections and evaluate the association between serum tumor marker levels (CEA, CA19-9, and CA72-4) and different status of *H. pylori* infections according to different *H. pylori* diagnostic tests.

## Materials and methods

### Study subjects, design and population

This cross—sectional study carried out by the collection of stool and blood samples from two hundred (200) patients, who have *H. pylori* infections from October 2019 to November 2020. All patients had been diagnosed by detection of serum *H. pylori* antibodies one-step immunochromatography test and confirmed by serum *H. pylori* IgG antibody to indicate chronic infection and SAT to indicate acute infection at some Hospitals, Gastroenterology, and Hepatology Clinics in Ibb governorate, Yemen. The patient's questionnaire was designed as demographic information of patients including age, gender, smoking, chewing Khat plant (*Catha edulis*), diet, education levels, fruit and vegetables eating, tea and coffee drinking, weight, height, family history of *H. pylori* infections and peptic ulcer disease (PUD), duration of *H. pylori* infection, presence and severity of gastrointestinal symptoms and treatment. Five to ten ml of venous blood sample was collected from patients.

### *H. pylori* testing and tumor markers measurement

Serum samples separated from whole blood divided and kept into two sterile Eppendorf tubes. The first separated serum tube was used to *H. pylori* immunochromatographic antibody test and serum *H. pylori* IgG antibody by Enzyme immunoassay (ELISA) test. The second separated serum was tested in Cobas e 411 analyzer (Roche Diagnostic, Hitachi, Japan) with Electro-Chemiluminescence Immunoassay (ECLIA) technique for detecting serum tumor markers (CEA, CA19-9 and CA72-4). In addition, stool samples collected from all patients for detection of *H. pylori* stool antigen and performed immediately. Furthermore, the body mass index (BMI) was calculated.

### Statistical analysis

The data were analyzed by a Statistical Package of Social Sciences (SPSS version 21). The results presented as percentages, means, standard deviations (SD). Also, *Chi*-square *X*^*2*^ test was used for categorical variables. Furthermore, comparison was made by sample independent t-test and one-way ANOVA. Correlation and simple linear regression were performed for scales variables. *P* values < 0.05 were considered as statistically significant**.**

## Results

The results of the demographic characterization of 200 patients with *H. pylori* infection showed that mean ± SD of the patient's age 36.80 ± 16.47; the majority of them were in the age group [20–39] years old. Out of all patients, 92 (46%) were males and 108 (54%) were females. 148 (74%) of the patients chaw the Khat (*Catha edulis*), 48 (24%) were smokers, 127 (63%) had family history of *H. pylori* infections and 60 (30%) of patients had family history of PUD. There were significant association (*P* < 0.05) between serum *H. pylori* IgG antibody and age groups, education levels, chewing Khat (*Catha edulis*), smoking, family history of *H. pylori* infections and PUD, recurrent infection, treatments and BMI. However, there was no significant association with residency, gender, vegetables and fruits eating and coffee and tea drinking. While the results of SAT showed significant association at (*P* < 0.05) with age groups, education levels, vegetables and fruits eating, recurrent infection and treatments. On the other hand, there was no significant association with residency, gender, coffee and tea drinking chewing Khat (*Catha edulis*), smoking, family history of *H. pylori* infections and PUD, recurrent infection and BMI (Table 1).

**Table 1:** Demographic characteristics of *H. pylori* patients and its association with *H. pylori* infection diagnostic tests

Variable	No./%	*H. pylori* IgG titer mean ± SD	*P* value	SAT		*X*^2^	*P* value
				Positive	Negative		
*Residency*							
Rural	110/55	24.46 ± 10.27	0.095	64	46	1.72	0.192
Urban	90/45	22.20 ± 8.49		44	46		
*Gender*							
Male	92/46	22.80 ± 9.24	0.376	53	39	0.89	0.345
Female	108/54	24.00 ± 9.826		55	53		
*Age group (years)*							
< 20	23/11.5	17.29 ± 9.84	< 0.001*	16	7	8.5	0.005*
20–39	104/52	21.37 ± 8.74		49	55		
40–59	47/23.5	27.02 ± 8.38		22	25		
˃ 59	26/13	30.72 ± 8.06		21	5		
*Education level*							
Elementary	105/52.5	25.12 ± 10.10	0.041*	61	44	3.35	0.187
High school	54/27	21.77 ± 8.53		30	24		
Professional	41/20.5	21.71 ± 8.41		17	24		
*Vegetables and fruit eating*							
Yes	172/86	22.97 ± 9.41	0.083	98	74	4.38	0.036*
No	28/14	26.34 ± 10.08		10	18		
*Tea and coffee*							
Yes	195/97.5	23.42 ± 9.40	0.821	106	89	0.4	0.525
No	5/2.5	24.40 ± 15.85		2	3		
*Khat (Catha edulis) Chewing*							
Yes	148/74	29.29 ± 9.44	0.035*	82	66	0.45	0.501
No	52/26	21.05 ± 9.55		26	26		
*Smoking*							
Yes	48/24	26.92 ± 8.37	0.004*	27	21	0.12	0.72
No	152/76	22.35 ± 9.66		81	71		
*Family history of H. pylori*							
Yes	127/63.5	25.91 ± 9.04	< 0.001*	68	59	0.02	0.864
No	73/36.5	19.16 ± 8.93		40	33		
*Family history of PUD*							
Yes	60/30	28.40 ± 9.06	< 0.001*	33	27	0.03	0.853
No	140/70	21.32 ± 8.98		75	65		
*Treatment*							
Yes	109/54.5	26.01 ± 9.00	< 0.001*	51	58	5.01	0.025*
No	91/45.5	20.38 ± 9.33		57	34		
*Recurrent infection*							
Yes	89/44.5	29.56 ± 8.84	< 0.001*	58	31	8.05	0.005*
No	111/55.5	18.54 ± 6.92		50	61		
*BMI*							
Underweight	27/13.5	22.98 ± 12.09	0.01*	17	10	2.29	0.502
Normal	94/47	22.09 ± 8.80		50	44		
Overweight	71/35.5	25.60 ± 8.76		35	36		
Obese	8/4	30.40 ± 8.65		5	3		

Data represented as mean ± SD, frequency percent%, *Chi* square test, Independent t test*,* one way AOVA *H. pylori*: *Helicobacter pylori*, SAT: stool antigen test and BMI: body mass index

*P < 0.05 considered significant

**Fig. 1:**
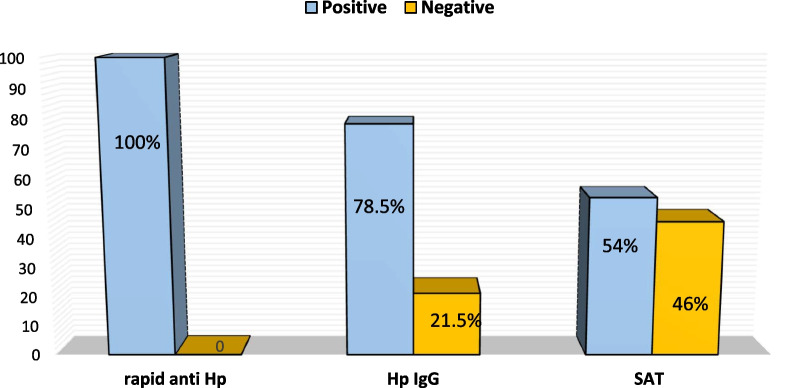
The prevalence of *H. pylori* infections by different tests

In addition, the findings showed that the prevalence of *H. pylori* infections by rapid anti *H. pylori* test were 200 (100%), 157 (78.5%) by serum *H. pylori I*gG antibody and 108 (54%) by SAT (Fig. 1).

**Fig. 2:**
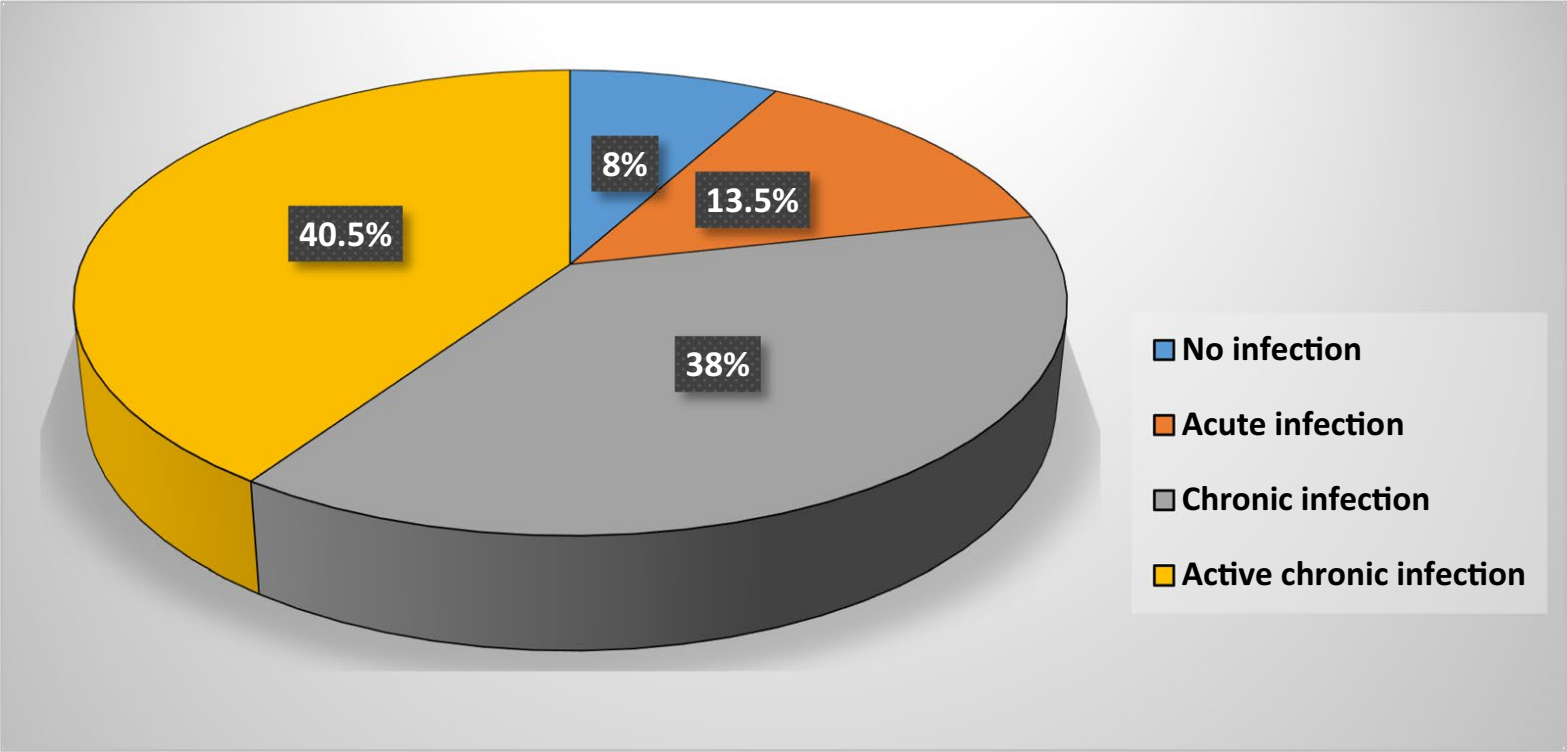
Classification of different stages of *H. pylori* infections

Clinically, the findings of the present study demonstrated the classification of different stages of *H. pylori* infections. 16 (8%) of the patients were negative for confirmatory tests by both serum *H. pylori* IgG antibody and SAT (no infections), 27 (13.5%) were only positive for SAT (acute infections), 76 (38%) were only positive for serum *H. pylori* IgG antibody test (chronic infections) and 81 (40.5%) were positive for both serum *H. pylori* IgG antibody and SAT (active chronic infections) (Fig. 2).

Interestingly, the results demonstrated that 42 (21%) of the patients had abnormal level of CEA, 30 (15%) had abnormal level of CA19-9 and 31(15.5%) had abnormal level of CA72-4. In addition the Table 2 show mean and SD of tumor markers for patients.

**Table 2:** Serum levels of some tumor markers among patients with *H. pylori* infections

Tumor markers	Mean ± SD	Normal (Within the normal range)		Abnormal (Out of the normal range)	
		Frequency	Percent%	Frequency	Percent%
CEA Normal range <5 ng/ml	2.92 ± 2.18	158	79	42	21
CA 19-9 Normal range <37 ng/ml	20.32 ± 14.43	170	85	30	15
CA 72-4 Normal range <6.9 ng/ml	3.72 ± 2.57	169	84.5	31	15.5

Data represented as mean ± SD, frequency percent%, CEA: carcinoembryonic antigen, CA19-9: cancer antigen 19-9 and CA72-4: cancer antigen 72-4

**Fig. 3:**
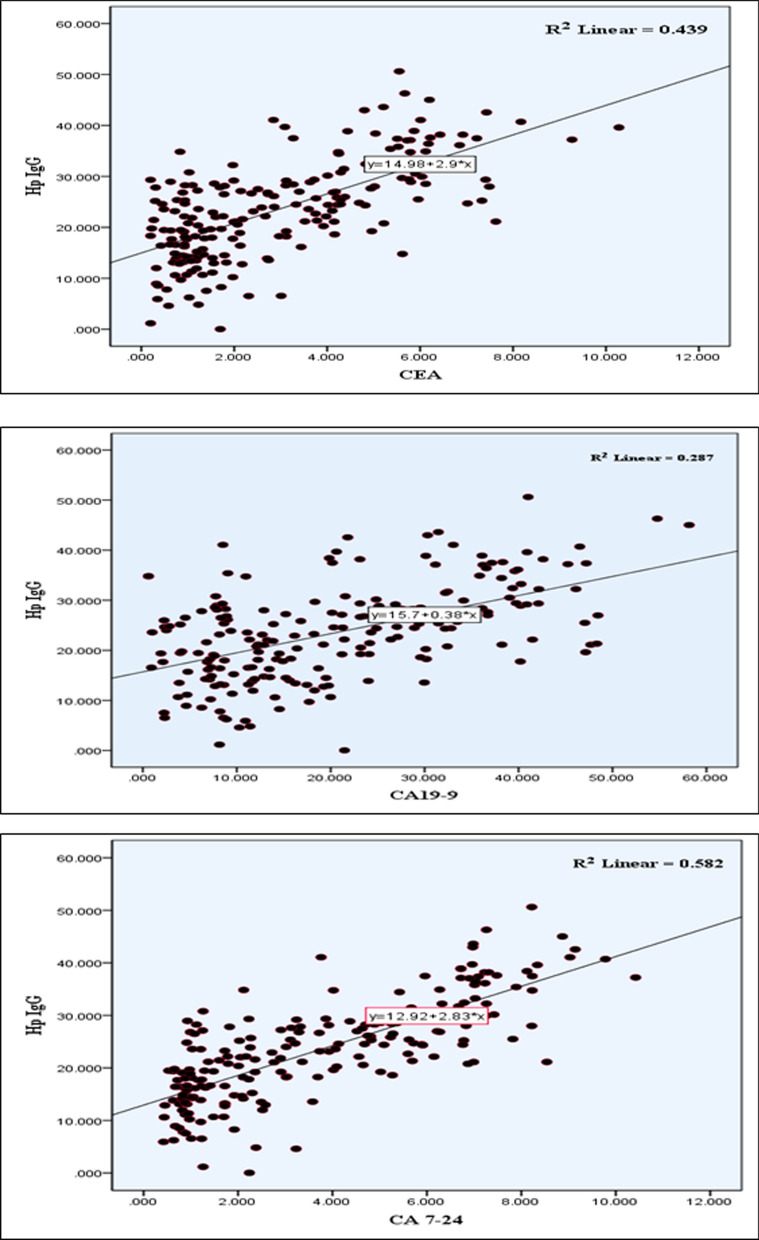
The correlation between serum *H. pylori* IgG antibody and tumor markers

Clinically, the data showed that there was statistical significant positive correlation between H. pylori IgG antibody and tumor markers (CEA, CA19-9 and CA72-4) which was measured by correlation coefficient and linear regression with B = 14.97, 15.70 and 12.91 and r^2^ = 0.439, 0.287 and 0.582 for CEA, CA19-9, CA72-4 respectively, (Table 3) and (Fig. 3). Additionally, the (mean ± SD) of tumor markers level (CEA, CA19-9 and CA72-4) were significantly increased in patients with SAT positive (*P *< 0.05), as show in (Table 4).

**Table 3:** Correlation between serum levels of tumor markers and serum *H. pylori* IgG Antibody

Tumor markers	*H. pylori* IgG		
	B	r^2^	*P* value
CEA	14.97	0.439	<0.001*
CA 19–9	15.70	0.287	<0.001*
CA 72–4	12.91	0.582	<0.001*

Data represented by Correlation coefficient and linear regression, CEA: carcinoembryonic antigen, CA19-9: cancer antigen 19-9, CA72-4: cancer antigen 72-4 and *H. pylori*: *Helicobacter pylori*, *P < 0.05 considered significant

**Table 4:** Association between serum levels of tumor markers and stool antigen test

Variable		SAT		*P* value
		Positive	Negative	
CEA	Mean ± SD	3.34 ± 2.39	2.43 ± 1.79	<0.001*
CA19-9	Mean ± SD	22.96 ± 14.5	17.22 ± 11.27	0.002*
CA72-4	Mean ± SD	4.39 ± 2.74	2.93 ± 2.12	<0.001*

Data represented by independent sample t test CEA: carcinoembryonic antigen, CA19-9: cancer antigen 19-9, CA72-4: cancer antigen 72-4 and SAT: stool antigen test, *P < 0.05 considered significant

Importantly, the findings showed the association between serum tumor markers levels and different age groups. There was statistically significant difference between serum tumor markers levels and different age groups at (*P* < 0.05) represented by mean ± SD for CEA, CA19-9, CA72-4, which was in the age group more than 59 years old were significantly higher than other groups. In addition, there was statistically significant increase in serum tumor markers levels (CEA, CA19-9, CA72-4) at (*P* < 0.05) as in symptomatic patients in comparison to non-symptomatic ones Tables 5 and 6.

**Table 5:** Comparison of serum tumor markers levels among different age groups

Age group (years)	N	Tumor markers					
		CEA	*P* value	CA19-9	*P* value	CA7-24	*P* value
		Mean ± SD		Mean ± SD		Mean ± SD	
< 20	23	1.50 ± 1.27	<0.001*	16.56 ± 11.96	<0.001*	2.27 ± 1.88	<0.001*
20–39	104	2.21 ± 1.83		15.45 ± 11.49		2.69 ± 2.06	
40–59	47	3.62 ± 1.88		26.24 ± 13.14		4.97 ± 2.19	
˃ 59	26	5.76 ± 1.72		32.43 ± 10.84		6.86 ± 1.96	

Data represented by one-way ANOVA, CEA: carcinoembryonic antigen, CA19-9: cancer antigen 19-9 and CA72-4: cancer antigen 72-4, *P < 0.05 considered significant

**Table 6:** Comparison of serum tumor markers levels with symptoms

Variable	Tumor Markers (Mean ± SD)		
	CEA	CA 19-9	CA 72-4
Symptomatic patients	3.02 ± 2.22	20.94 ± 13.75	3.89 ± 2.62
Asymptomatic patients	1.96 ± 1.50	14.39 ± 7.91	2.11 ± 1.21
*P* value	0.043*	0.043*	0.004*

Data represented by one-way ANOVA, CEA: carcinoembryonic antigen, CA19-9: cancer antigen 19-9 and CA72-4: cancer antigen 72-4, *P < 0.05 considered significant

Finally, the results indicated that the serum tumor markers levels were associated with different *H. pylori* infection status. There were a significantly increased in active chronic infection group as (mean ± SD) of CEA (4.02 ± 2.34), CA19-9 (26.44 ± 14.73), and CA72-4 (5.28 ± 2.49) than other patients groups (*P *< 0.05). In addition, the results indicated that there were statistically significant difference of serum tumor markers levels based on infection duration, the (mean ± SD) of serum tumor markers levels CEA (3.99 ± 2.16) CA19-9 (26.86 ± 13.52), and CA72-4 (4.850 ± 2.806) respectively, which were the highest in the group with more than 3 years of *H. pylori* infection (Table 7) and (Table 8).

**Table 7:** Comparison of serum tumor markers levels among different *H. pylori* infection status

*H. pylori* infection status	N	Tumor markers					*P* value
		CEA	*P* value	CA-19–9	*P* value	CA-72–4	
		Mean ± SD		Mean ± SD		Mean ± SD	
No infection	16	1.20 ± 0.680	<0.001*	13.62 ± 6.09	<0.001*	1.42 ± 0.98	<0.001*
Acute infection	27	1.44 ± 1.15		12.52 ± 7.33		1.74 ± 1.43	
Chronic infection	76	2.64 ± 1.85		17.98 ± 11.39		3.25 ± 2.16	
Active chronic infection	81	4.02 ± 2.34		26.44 ± 14.73		5.28 ± 2.49	

Data represented by one-way ANOVA, CEA: carcinoembryonic antigen, CA19-9: cancer antigen 19-9 and CA72-4: cancer antigen 72-4, *P < 0.05 considered significant

**Table 8:** Comparison of serum tumor markers levels among different *H. pylori* infection duration

*H. pylori* infection duration	N	Tumor markers					
		CEA	*P* value	CA199	*P* value	CA724	*P* value
		Mean ± SD		Mean ± SD		Mean ± SD	
1 month	51	2.18 ± 2.12	0.001*	16.14 ± 12.19	<0.001*	2.70 ± 2.36	<0.001*
6 months	41	2.85 ± 2.05		17.55 ± 13.66		3.44 ± 2.45	
1–3 years	63	2.81 ± 2.08		20.83 ± 12.56		3.92 ± 2.34	
˃ 3 years	45	3.99 ± 2.16		26.86 ± 13.52		4.85 ± 2.80	

Data represented by one-way ANOVA, CEA: carcinoembryonic antigen, CA19-9: cancer antigen 19-9 and CA72-4: cancer antigen 72-4, *P < 0.05 considered significant

## Discussion

Yemen is a developing country with high seroprevalence of *H. pylori* infections which is the most common infection of about 75–82.2%. These high percentages are due to the lack of proper sanitation, lack of safe drinking water, bad hygienic habits, eating of vegetables and chewing Khat (*Catha edulis*) without improper washing or contamination of sewage water through rinsing these plants in agriculture, poor diets and overcrowding of some Yemeni population [27, 28]**.**

The results of the current study, revealed that the incidence of *H. pylori* infections by rapid anti *H. pylori* test were (100%), (78.5%) by serum *H. pylori I*gG antibody and (54%) by SAT as it is shown in Fig. 1.

The finding of the current study were consistent with several previous studies such as [29] which reported that 48.3% of the study subjects tested positive for *H. pylori* IgG while 28.2% tested positive for SAT. Similarly a study conducted in Yemen [30] found that 72% of the participants tested positive for blood antibody test, and 49% tested positive for SAT.

Another study, [31] also supported the results of the current study by reporting a high positivity for serum antibodies compared to antigen in stool. Study [32] further supported the findings of the present study by reporting a seroprevalence of 78.4% for *H. pylori* infection using *H. pylori* serum IgG ELISA. In Oman, study [33] found a seroprevalence of 69.5% of *H. pylori* infection based on serum IgG ELISA in individuals aged 15–50 years. However, the results of the present study contradicted a study conducted by [34] in Kingdom Saudi Arabia which showed a prevalence of 28.3% for *H. pylori* using ELISA to detect serum *H. pylori* IgG antibody.

In the present study 54% of study subjects tested positive for SAT, which aligned with the findings of [35] which also reported a 54% positive rate of SAT. Additionally, [30] found that 49% of the participants were diagnosed as positive for *H. pylori* infection and 51% as negative based on SAT.

The detection of *H*. *pylori* stool antigen and the urea breath test (UBT) are reliable indicators for identifying ongoing (active) *H*. *pylori* infection; whereas serological testing of *H*. *pylori* IgG antibodies indicate a previous (past or long standing infection) as infection become more chronic, the shedding of *H*. *pylori* decreases which is often seen in the older individuals [36]. Another study by [37], showed that the gold standard for the diagnosis of *H. pylori* infection status was considered positive on the concordance of at least two different tests in other term the most accurate diagnosis of *H. pylori* infection is achieved when at least two different tests are used and show consistent results.

The study conducted by [38] found a lower rate of positive results for the SAT compared to the current study with a positivity rate of 45%. However the present study contraindicated the results of [39] who reported a SAT positivity rate 82% and [40] which found a SAT positivity rate of 32.22% in their study subjects. Another study by [3] highlighted that H. *pylori* infection remains a significant public health issue, with an estimated 4.4 billion individuals worldwide testing positive for *H. pylori*, the chronic *H. pylori* infection. This chronic infection is typically acquired during childhood.

The findings of the current study revealed a significant association (*P* < 0.05) between serum *H. pylori* IgG antibody level and various factors including age groups, education levels, chewing Khat (*Catha edulis*), smoking, family history of H. *pylori* infections and PUD, recurrent infection, treatments, and BMI values. However, no significant association were found with residency, gender, vegetables and fruit eating, or coffee and drinking tea. Additionally, the present study demonstrated significant associations (*P* < 0.05) between SAT results and age groups, education levels, consumption of vegetables and fruit eating, recurrent infection, and treatments. Conversely, no significant association were found with residency, gender, coffee and tea drinking, chewing Khat (*Catha edulis*), smoking, family history of *H. pylori* infections and PUD, recurrent infection, or BMI. These association are presented in Table 1.

The finding of this study were consistent with previous research [41], which found no significant difference in *H. pylori* infection between males and females (*P *˃ 0.05) this also supported by another study [42]. However, the finding of the current study contradict the finding of [43]**,** who a statistically significant association between seropositivity of *H*. *pylori* and gender. On the other hand, this study align with the findings of [44], which found a higher prevalence of *H. pylori* infection among patients who chewed Khat (*Catha edulis*). In contrast [45] didn’t find a significant difference in *H. pylori* infection based on Khat (*Catha edulis*) chewing. Several studies have suggested that smoking is a risk factor for various diseases including GC, as well as *H. pylori* infection [45–47]. The current study found that serum *H. pylori* IgG antibody levels were significantly higher in smokers compared to non-smokers (*P *< 0.05), which contradicts the findings of [48] who no significant difference in *H. pylori* infection between smokers and nonsmokers (*P *˃ 0.05).

In regarding residency, the results of the present study were in agreement with [49] who found no significant difference in *H. pylori* infection between patients from urban and rural areas (*P *˃ 0.05).

Additionally the current study revealed a positive correlation between serum *H. pylori* IgG antibody levels and BMI (*P *< 0.05), which is supported by previous studies that demonstrating an association between *H. pylori* infection and BMI [50–53].

Furthermore, the present study was consistent with a study conducted by [54] which found a significant association between *H. pylori* infection and history of PUD (*P *< 0.05). In contrast, the results of the current were disagreed with a study by [55] which found no statistical significant difference in *H. pylori* infection based on family history of PUD.

Chronic *H. pylori* infection is most commonly observed in the antrum of the stomach in developed countries, and it is usually linked to duodenal ulcers. In contrast, *H. pylori* infection in developing countries tends to be more localized in the corpus. In this case, *H. pylori* infection initiates a series of the cascade events including CG, gastric atrophy, intestinal metaplasia, dysplasia, and eventually invasive carcinoma [56, 57]**.**

Anti *H. pylori* antibody, CA724, CA19-9, and CEA are all markers for early GC screening. However, their levels and their correlation with clinicopathological features of young patients with early GC, as well as their role in evaluating postoperative recurrence, metastasis, and death have not been extensively studied [11]**.**

*H. pylori* infections is the most common cause of gastritis and gastritis is a precursor to GC. Many studies have reported that serum CEA levels increase in some benign diseases such as gastritis and there is a significant difference between patients diagnosed with gastritis before the development of GC [58–60].

Importantly, the results of the present study demonstrated that 42 (21%) of patients had abnormal level of CEA, 30 (15%) had abnormal level of CA19-9 and 31(15.5%) had abnormal level of CA72-4, as shown in Table 2. Furthermore, there was a positive correlation between serum *H. pylori* IgG antibody and tumor markers (CEA, CA19-9, CA72-4). Additionally, the results showed that tumor markers levels were significantly correlated with level of serum *H. pylori* IgG antibody with B = 14.97, 15.70, 12.91 and r^2^ = 0.439, 0.287, 0.582 for CEA, CA19-9, CA72-4 respectively. Additionally, there was statistically significant difference in tumor markers levels CEA, CA19-9 and CA72-4 based on SAT status, with higher levels observed in patients with SAT positive (*P *< 0.05) as shown in Tables 3, 4 and Fig. 3.

The current study findings were in agreement with [26] which found that subjects in the *H. pylori* infection group had significantly higher levels of CEA, alpha-fetoprotein (AFP) and CA7-24 compared to those in the *H. pylori* negative group**.** Another study [61], also reported a correlation between CEA level and *H. pylori* IgG levels where CEA levels were higher in patients who tested positive to *H. pylori* IgG compared to those who tested negative. Similar results were reported in other studies, which demonstrated elevated levels of CEA in the *H. pylori* infected group [11, 24, 25]. Serum CEA was also found to be significantly associated with anti- *H. pylori* antibody levels and severity of GC [11]**.**

In study conducted on adults, it was found that there is a significant positive correlation between serum CEA levels and *H. pylori* infection. Additionally, the study observed that serum CA 74-2 levels were elevated in individuals with *H. pylori* infection compared to those without the infection. This findings shed light on the association between *H. pylori* infection and the development of in the incidence of gastric, pancreatic, and lung cancers [26]. Another study [62] reported elevated levels of CA72-4 in patients with *H. pylori* infection, and the levels returned to normal after two courses of eradication therapy. Furthermore, another study [11] indicated that serum CA72-4 is associated to the presence of *H. pylori* infection.

The present study supported the previous study [63] that showed a relation between *H. pylori* infection related and elevated levels of CEA in the serum. Another study [64] found that tumor markers such as CEA, CA19-9 and AFP were prognostic factors for GC and that *H. pylori* infection is most common cause of GC. Additionally, a study [12] found that GC developed in patients with *H. pylori* infection but not in those without the infections.

In addition, [65], who reported that CEA, CA19-9 were high in benign gastric disease, these result was in agreement with many previous results which showed the correlation between *H. pylori* gastritis and levels of these tumor markers and compare the value of four tumor markers (CEA,CA72-4, CA19-9 and CA125) were increased in early GC.

As well as study of [66] reported the association between level of CEA and CA19-9 among benign gastric disease with high mean of both tumor markers, this means that benign gastric disease like gastritis may lead to increase levels of serum tumor markers and thus can be used to know the possible development of *H. pylori* infection to GC.

The results of our study with previous studies confirmed the association between *H. pylori* infection and several varieties of cancer, which proved from positive associations between *H. pylori* infection and the development of GC [67–69].

Another study [70] reported that high CA72-4 value is correlated with GU. In addition, the long duration of *H. pylori* infections and high CEA value are positively associated with high CA72-4 levels.

Furthermore, the findings of the present study found that serum level of CEA, CA19-9 and CA72-4 was significantly higher among *H. pylori* infected patients with SAT positive, and it might develop into GC as reported by many studies that atrophic gastritis is a well-recognized precancerous lesion, and it might develop into GC [59, 71–73]. In addition [74], reported that there was increase in CEA and CA19-9 in *H. pylori* positive patients in comparison to *H. pylori* negative, this may indicate with increase inflammatory process due to *H. pylori* infections; the level of tumor markers mostly increased. Other previous study showed that acute *H. pylori* infection also causes elevated levels of CEA, indicating that in GC suspected patients the influence of inflammation on CEA levels should not be excluded [24, 75]. In the other study [61] reported that CEA level was correlated to the level of *H. pylori* IgG where that CEA level was higher in patients who were positive to *H. pylori* IgG in comparison to negative patient. In addition our study was in agreement with [63] who indicated that *H. pylori* infection was related to the level of CEA in the host serum. Meng et al. [64] found that tumor markers including CEA, CA19-9, and AFP were demonstrated to be prognostic factors for GC. In addition, [12] found that *H. pylori* is most common cause of GC; this fact indicated by that GC developed in patients with *H. pylori* infection than non-infected ones.

Interestingly, the current study found a significant association between serum tumor markers levels and different age groups with a statistically significant difference observed (*P* < 0.05) as shown in Table 5. In addition, there was a statistically significant increase in serum tumor markers levels CEA, CA19-9 and CA72-4 among symptomatic patients in comparison to non-symptomatic ones, at (*P* < *0.05*) as shown in Table 5.

The results of this study were in agreement other studies which report that CEA level was significantly associated with age (*P *< 0.05) [42, 76].

The finding of this study showed that serum tumor markers levels were associated with different* H. pylori* infection status. Furthermore, serum levels of tumor markers CEA, CA19-9 and CA72-4 were significantly higher in patients with active chronic infection (positive for both serum *H. pylori* IgG antibody and SAT) compared to other groups at (*P *< 0.05) as shown in Table 7. In addition, there was statistically significant difference of serum tumor markers levels based on infection duration, as shown in Table 8.

Many previous studies, including the case control studies have shown an increase in serum levels of tumor markers in the development of GC in *H. pylori* infected individuals and increasing with the long duration of seropositivity. Serum *H. pylori* IgG antibodies indicated to chronic *H. pylori* infection, the current study found that there was relation between chronic *H. pylori* infections and serum tumor markers at Ibb governorate. The patients with *H. pylori* infections had a higher CEA, CA19-9, CA72-4 levels and the levels of these markers usually significantly associated with *H. pylori* infection status. These results were corresponded with many previous studies, which have shown an increase odds ratio for the development of GC in *H. pylori* infected individuals, it is also associated with the duration of seropositivity of *H. pylori* IgG [77–79].

Same results also was obtained by [80] who reported that patients with *H. pylori* infection have significantly higher levels of CEA, AFP and CA724 than those in the *H. pylori* negative group. Further studies focusing on the association between *H. pylori* infection and cancer risks illustrated that *H. pylori* infection might be an independent carcinogenic risk factor. The one of the possible mechanisms of oncogenic transformation is *H. pylori* infection that triggers inflammation resulting in interactions between the bacteria and host cells in local and distant microenvironments [81]**.**

Contributions of the current study. First: it is the first study in Ibb governorate, Yemen to diagnose *H. pylori* infection using various tests to identify the different types of *H. pylori* infection; chronic, acute and active chronic infection. Second: it is the first study in Yemen to determine the correlation between serum *H. pylori* IgG antibody and serum level of more than one tumor markers including CEA, CA19-19 and CA72-4.

## Limitations

Two important limitations need to be acknowledged and referred to hence. First: due to the time limit and the economic situation in Yemen, the researchers administered the study instruments to (200) patients. Second: there are many difficulties in obtaining the samples and testing it. These obstacles includes war, siege and COVID-19.

In addition to the previously described situations, the researchers hoped to obtain the requirements for diagnosis *H. pylori* infection based on UBT and molecular diagnostic methods like polymerase chain reaction. However, the situation of our country and the critical stage of the world at that time prevented us from obtaining it. Consequently we used the SAT and serum *H. pylori* IgG antibodies.

## Conclusions

Based on the findings the conclusions of the current study indicated that the serum levels of tumor markers were mostly significant and associated with chronic *H. pylori* infections. In addition, the findings confirmed that the level of serum tumor markers (CEA, CA19-9 and CA72-4) were associated with the levels of IgG antibody and positivity of SAT to *H. pylori* infections. The patients with active chronic *H. pylori* infections have increased of serum levels of tumor markers. Thus, consequence may be associated with GC in these patients.

## Data Availability

The data are not publicly available due to privacy or ethical consideration. The corresponding can provide the data that support the findings of the study upon request.

## Abbreviations

AFP: Alpha-fetoprotein
CA19-9: Cancer antigen 19-9
CA72-4: Cancer asntigen 72-4
CagA: Cytotoxin associated gene
CEA: Carcinoembryonic antigen
CG: Chronic gastritis
ECLIA: Electro-chemiluminescence immunoassay
ELISA: Enzyme immunoassay
GC: Gastric cancer
GU: Gastric ulcer
H. pylori: Helicobacter pylori
PU: Peptic ulcer
PUD: Peptic ulcer disease
SAT: Stool antigen test
SD: Standard deviation
SPSS: Statistical package of social science program
UBT: Urea breath test
VacA: Vacuolating cytotoxin A
WHO: World Health Organization
